# Endurance Effort Affected Expression of Actinin 3 and Klotho Different Isoforms Basing on the Arabian Horses Model

**DOI:** 10.3390/genes15121618

**Published:** 2024-12-18

**Authors:** Grzegorz Myćka, Katarzyna Ropka-Molik, Anna Cywińska, Monika Stefaniuk-Szmukier

**Affiliations:** 1 Department of Animal Molecular Biology, National Research Institute of Animal Production, Krakowska 1 Street, 32-083 Balice, Poland; grzegorz.mycka@iz.edu.pl (G.M.); monika.stefaniuk@iz.edu.pl (M.S.-S.); 2 Faculty of Biological and Veterinary Sciences, Nicolaus Copernicus University in Torun, Lwowska 1 Street, 87-100 Torun, Poland; anna_cywinska@umk.pl

**Keywords:** *ACTN3*, *KL*, Arabian horse, endurance, RNA

## Abstract

Background: Among numerous genes that have been a focus of equine genetic research, the *KL* (Klotho) and *ACTN3* (Alpha-actinin-3) genes stand out due to their significant roles in muscle function and overall health, as well as performance ability. Previous studies on Arabian horses and other mammalians have shown that both *KL* and *ACTN3* occur in different isoforms that seem to have different roles in metabolism. The main purpose of this present study was to describe different isoforms (*ACTN3*, *ACTN3-201*, *ACTN3-202*, *KL*, *KL-202*, *KL-203*) expression levels affected by the endurance effort in Arabian horses. Methods: Blood samples were taken from a group of *n* = 10 Arabian horses taking part in a long-distance 120 km endurance ride. After RNA isolation and reverse transcription, real-time PCR was performed. The expression levels (Relative Quantity, RQ) were calculated using the delta-delta CT method. The results showed surprisingly large differences between different isoforms expression levels which brought us to the conclusion that both *KL* and *ACTN3* genes are suitable genetic markers to measure endurance performance. Moreover, the correlation network analyses showed that the *MIOX* (myo-inositol oxygenase), *SH3RH2* (SH3 domain-containing ring finger 2) and *TNNI2* (Troponin I2, fast skeletal type) genes are significantly involved in the endurance effort metabolism.

## 1. Introduction

The analysis of Arabian horses exercise ability offers a unique opportunity to understand how specific gene variants can influence the equine endurance athleticism and overall performance. Endurance capacity is a specific example of a sport phenotype determined by physiological and genetic factors. Recent studies have shown that alternative splicing can be understood as an evolutionary adaptation to many factors occurring in an individual’s life [1]. Moreover, a study by Innocenzi et al. (2021) [2], based on transcriptome analyses, confirmed the occurrence of alternative splicing induced by aerobic exercise in a mice model. In humans, it has been shown that physical exercise can modify different isoforms of the genes encoding proteins crucial for skeletal muscle contraction [3].

Among numerous genes that have been a focus of equine genetic research, the *KL* (Klotho) and *ACTN3* (Alpha-actinin-3) genes stand out due to their significant roles in muscle function and overall health, as well as performance ability. The *KL* gene, known for its involvement in the regulation of aging and calcium homeostasis, has been implicated in longevity and physical performance in various species, including humans [4]. In mammals, the function of *KL* has been studied and is known for its involvement in modulating aging, phosphate metabolism and calcium homeostasis, as well as for its protective roles against oxidative stress and inflammation [5,6]. Recent studies have shown that in horses, *KL* plays a crucial role in longevity and is considered a genetic marker for exhaustion of the organism [7]. Importantly, the occurrence of various *KL* gene isoforms has been confirmed in the mammalian genome [8]; however, its presence and role in equines have not been studied so far.

On the other hand, the *ACTN3* gene, which encodes a protein essential for fast-twitch muscle fiber function, has been extensively studied in humans, with specific variants correlating with athletic performance [9,10]. In sports genetics, this gene is used to determine the sports predisposition of an athlete, considering sprinters and marathon runners [11]. Its role in equine muscle physiology and performance, especially in a breed as specialized as the Arabian, is of significant interest and has been widely described. The studies of Ropka-Molik et al. (2017) [12] and Ropka-Molik et al. (2019) [13] have shown the exercise-induced modification of *ACTN3* expression, resulting in decreases in transcript levels as a response to the intensive physical effort. Moreover, the regulation of two different *ACTN3* gene isoforms has strongly indicated their different roles in the equines’ metabolism during the effort.

Based on previous high-throughput genetic analyses, the present study aims to investigate the prevalence and impact of splice variants of the *KL* and *ACTN3* genes based on Arabian horse endurance effort. The presented findings shed light on the genetic underpinnings that may contribute to the breed’s renowned endurance and athleticism. Furthermore, it provides insights into the evolutionary aspects of equine athleticism, offering perspectives on how these genes have contributed to the distinct characteristics of the Arabian breed.

## 2. Materials and Methods

### 2.1. Animals and Gene Selection

Blood samples were taken from 10 Arabian horses (5 mares and 5 geldings, not related, 7–13 years old; average age 9.6 ± 2.2 years) 1 h before and 1 h after the end of the 120 km endurance ride under the supervision of the President of the Veterinary Commission and the approval of the President of Ground Jury (the year of the ride—2018). The exact riding schedule, sample collection and maintenance of the horses are described in detail by Myćka et al. (2024) [7]. Blood samples were taken via jugular venipuncture into a Tempus™ Blood RNA Tube (Applied Biosystems, Thermo Fisher Scientific, Waltham, MA, USA) and stored at −20 °C. Based on previous reports, the *ACTN3* [12] and *KL* [7] genes have been selected for analyses.

### 2.2. RNA Isolation

RNA was isolated from whole blood using the MagMAX™-96 total RNA isolation kit (Invitrogen, Thermo Scientific, Waltham, MA, USA) with a magnetic bead-based purification method according to the manufacturer’s protocol. Finally, a TapeStation 2200 (RNA ScreenTapes, Agilent Technologies, Santa Clara, CA, USA) and a Nanodrop 2000 spectrophotometer (Thermo Scientific, Waltham, MA, USA) were used to validate the sequence and quality, respectively, of the collected genetic material. The samples with a concentration higher than 100 ng/µL and a RNA integrity number (RIN) above 7.5 were used for further analyses.

### 2.3. Real-Time PCR Gene Expression Measurements

A total of 250 ng of total RNA was reverse transcribed to cDNA using a high-capacity RNA-to-cDNA kit (Applied Biosystems, Thermo Fisher Scientific, Waltham, MA, USA) in accordance with the manufacturer’s protocol. Primers were designed using the Primer3.0 freeware to amplify both the *ACTN3* and *KL* genes and their two isoforms, while primers for the endogenous control, the *B2M* gene, were described previously by Knych et al. (2016) [14] (Table 1). The exact transcript level was analyzed using the real-time PCR method on QuantStudio 7flex (Applied Biosystems, Thermo Fisher Scientific, Waltham, MA, USA). The reactions for each sample and for each gene included the endogenous control and were carried out in 3 replicates using EvaGreen^®^ qPCR Mix Plus (ROX) (Novazym, Poznan, Poland) according to the protocol. All reactions for each gene for all horses were performed on one plate to avoid batch effect. The efficiency of the PCR reaction was estimated based on the standard curve method, which was then used for the calculation of *ACTN3* and *KL* expression levels (Relative Quantity, RQ) based on the delta-delta CT method.

### 2.4. Data Analysis

The mean values of the Relative Quantity (RQ) were calculated for each variant separately using the SAS Enterprise Guide 8.3 software. For comparison between groups, the non-parametric U-Mann–Whitney test was performed for every isoform for a *p*-value confidence interval of 0.05. The String v12.0 software with Equus caballus (EcuCab3.0) reference was applied to identify the interaction between *ACTN3*, *KL* and other genes in the horse genome.

## 3. Results

### 3.1. Differences in ACTN3 Transcript Expression Level in the Context of Endurance Ride

**Figure 1:**
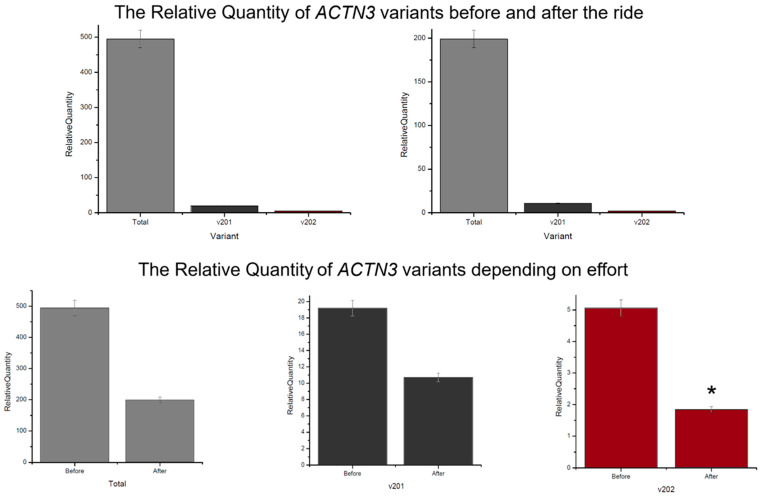
The Relative Quantity (RQ) of *ACTN3* gene variants, *—*p*-value < 0.05 for U-Mann–Whitney test. The RQ values were calculated relative to the endogenous control *B2M* gene.

**Figure 2:**
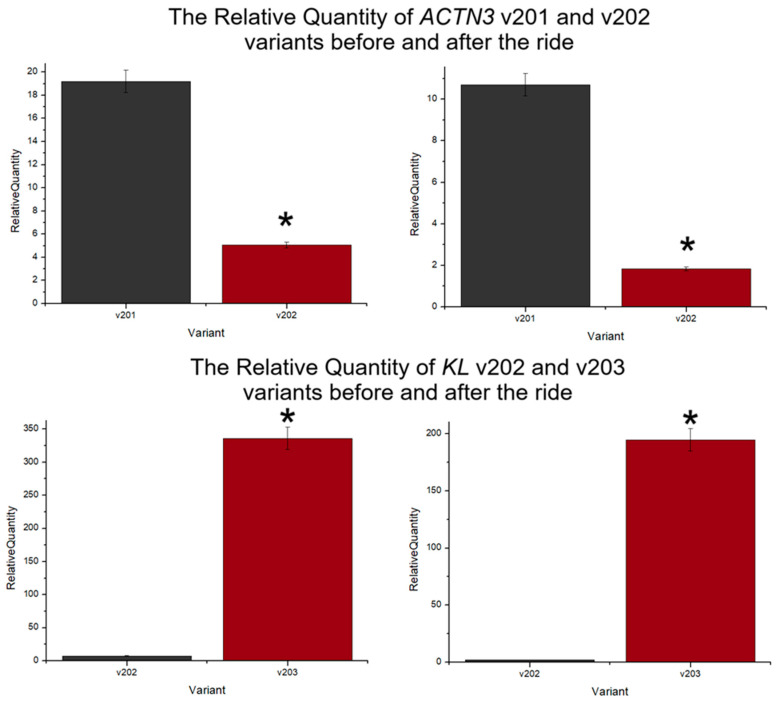
The Relative Quantity of *ACTN3* and *KL* variants before and after the 120 km ride, *—*p*-value < 0.05 for the U-Mann–Whitney test. The RQ values were calculated relative to the endogenous control *B2M* gene.

The ACTN3 expression level for each analyzed isoform decreased after the ride: variant total—from 494.81 to 226.05; v201—from 19.19 to 10.71; v202—from 5.06 to 1.84. The highest change, 2.75-fold, and significant downregulation were detected in v202 (Figure 1). Moreover, the percentage expression share of the v202 splice variant was the lowest among all analyzed. Such a small contribution of v201 and v202 compared to the total expression of ACTN3 gene suggests the presence of some other, previously unknown, transcription variants. Excluding the physical effort factor and comparing the expression of both splice variants, a significantly higher transcript level was observed for the v201 isoform. The obtained differences between isoforms were as follows: 3.8-fold and 5.8-fold higher expression level of v201 compared to v202 despite the measurement time (Figure 2).

### 3.2. Differences in KL Transcript Expression Level in the Context of Endurance Ride

**Figure 3:**
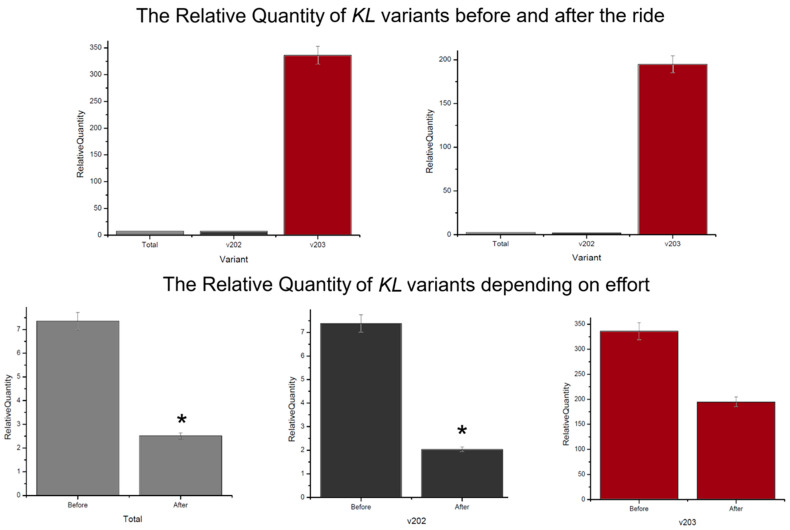
The Relative Quantity of *KL* gene variants, *—*p*-value < 0.05 for U-Mann–Whitney. The RQ values were calculated relative to the endogenous control *B2M* gene.

The *KL* expression levels in the context of endurance effort showed the same trend for each amplicon and were lower for each analysed variant after the ride, respectively: variant total—7.35 before and 2.51 after, v202—7.38 and 2.03, v203—336.04 and 194.76. Significant differences were obtained for the total KL expression and v202 (*p*-value < 0.05) (Figure 3). The comparison of the expression levels of two *KL* isoforms indicated that v203 isoform was significantly more abundant compared to v202 in both measurement time—before and after the ride: 45.5-fold and 96-fold; respectively (Figure 2).

### 3.3. Molecular Interaction Network Between ACTN3, KL and Most Related Genes

**Figure 4:**
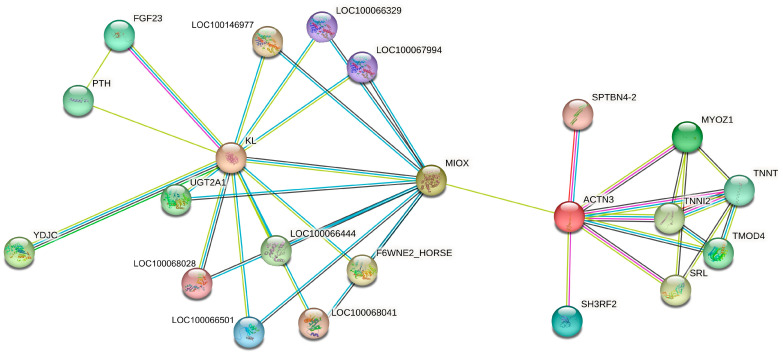
The correlation between *ACTN3* and *KL* with the most closely related genes included according to String software (v12.0) and based on Equus caballus reference.

In order to identify the broader perspective of the impact of the studied genes on exercise phenotype, the interaction network based on String software v12.0 and Equus caballus reference was created (Figure 4). The molecular relationship of up to 20 connected genes indicated that the most related genes interacted with the core-analyzed *KL* and *ACTN3* genes. Interestingly, the *MIOX* gene was identified as a molecular factor connecting both analyzed genes. Moreover, some genes (such as *SH3RH2* and *TNNI2*) (Figure 4), belonging to the *KL*–*ACTN3* molecular network, were previously identified as related to sport phenotype in horses [15] and in other mammals [16].

**Figure 5:**
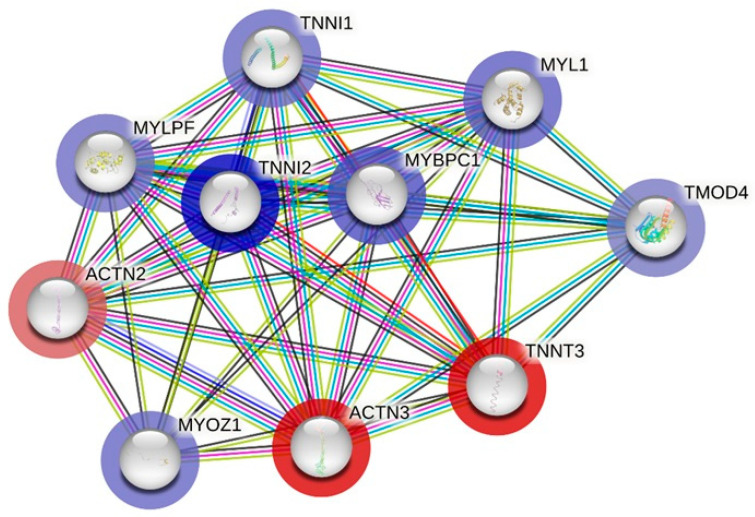
The interaction between *ACTN3* and the most closely related genes according to String software (v12.0) and based on Equus caballus reference with a confidence threshold set at 0.400 (the maximum number of interactions up to 20). Upregulation—blue halo; downregulation—red halo. Color gradient represents increasing FC value based on previously obtained data, accession number GSE226819.

**Figure 6:**
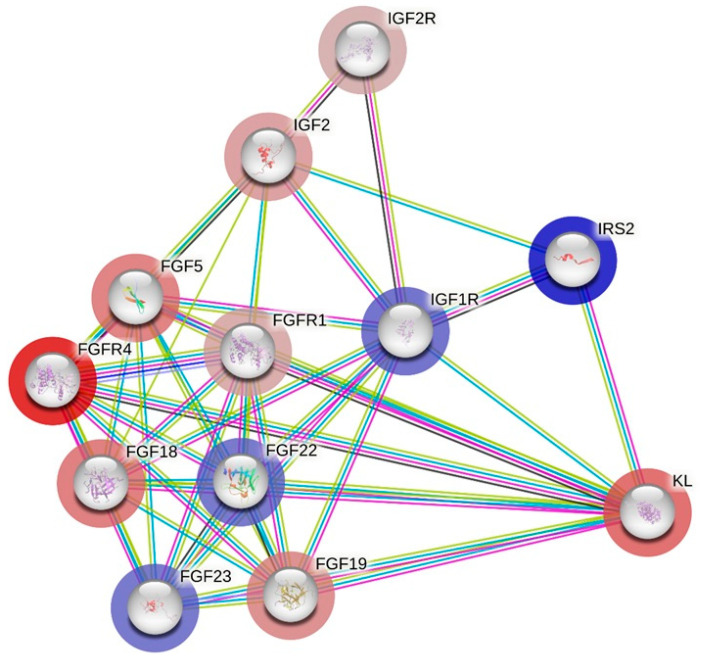
The interaction between *KL* and the most closely related genes according to String software (v12.0) and based on Equus caballus reference with a confidence threshold set at 0.400 (the maximum number of interactions up to 20). Upregulation—blue halo; downregulation—red halo. Color gradient represents increasing FC value based on previously obtained data, accession number GSE226819.

Next, for these genes, the fold change (FC) values corresponding to the differences in expression in blood under endurance effort in Arabian horses were assigned (GEO accession number GSE226819; [7]) (Figure 5 and Figure 6). The separate molecular networks considering *ACTN3* (Figure 5) and *KL* (Figure 6) with background genes were generated. The *ACTN3* gene was closely associated with the upregulation of *MYOZ1*, *TNNI1*, *TNNI2*, *MYBPC1*, *MYL1* and *TMOD4* and the downregulation of *ACTN2* and *TNNT3*. The *KL* gene demonstrated the closest association with the upregulation of *IRS2*, *IGF1R*, *FGF22* and *FGF23* and the downregulation of *FGFR1*, *FGFR4*, *FGF5*, *FGF18* and *FGF19*.

## 4. Discussion

Genes responsible for adaptation to exercise, especially endurance exercise, which are unique and require the involvement of many processes, are still being searched for. Based on previous research in horses, the candidate genes included the *ACTN3* [13] and *KL* gene [7]. Both genes were considered to be involved in exercise effort in horses based on whole transcriptome sequencing of muscle or/and blood [7,13]. Moreover, their role in metabolism regulation (*ACTN3*, *KL*), the inhibition of oxidative stress and the controlling of Ca^2+^ and phosphate homeostasis [4] confirms the need to test both genes for their involvement in exercise-related responses in horses.

The *ACTN3* gene encodes α-actinin-3, the protein specifically expressed in type II (fast-twitch) muscle fibers designed for quick, forceful contractions, and is particularly prevalent in muscles involved in sprinting and other power activities with very high energy consumption. On the contrary, type I muscle fibers are characterized by slow twitch and are more efficient in aerobic effort. The study by Norman et al. (2014) [17] showed that the lack of functional α-actinin-3 is associated with reduced activity of glycogen phosphorylase and increased activity of glycogen synthase, as well as higher muscle glycogen content. The authors suggested that these changes may lead to a decreased capacity of muscle to use glycogen as a fuel and increased consumption of simple carbohydrates resulting in higher ATP production. The conclusion is that the presence or absence of α-actinin-3 due to the variations in *ACTN3* gene variants, along with its isoforms, significantly affects muscle function and athletic performance via metabolism modification. In human athletes, these findings showed the *ACTN3* gene as a genetic marker of sprinting or marathon effort [10].

Outside of human studies, some very promising results have also been obtained in equines. The downregulation of *ACTN3* gene was detected for the first time in Arabian horses during whole transcriptome analysis on muscle tissue during the training of young horses to flat races [12]. The significance of *ACTN3* extends into its isoforms that can result from gene expression modulation, alternative splicing or post-translational modifications, each potentially having distinct roles in muscle physiology. The decreased expression of both isoforms and total variant of the *ACTN3* gene after the endurance ride is in accordance with the downregulation described previously in Arabian horses that took part in flat racing [13].

The results of this study, conducted on Arabian horses that completed a 120 km ride, confirmed that the downregulation of the v202 isoform showed the highest rate, 2.75-fold, while the share of that isoform was the lowest. Such results may suggest that the protein encoded by the v202 variant has a limited role in muscle metabolism during endurance effort, much less than other α-actinin-3 isoforms. In comparison, the v201 downregulation rate was 1.80-fold and the expression level was higher than the v202 isoform in every case, but still represented a small share of the total variant. In the study by Almarzook et al. (2019) [18] the genetic variation in ACTN3 was investigated in terms of endurance exercise in Arabian horses. The authors suggested that the occurrence of a SNP 12:26524930T>C mutation in 3′UTR may significantly affect gene expression, and consequently, result in better predispositions of horses for endurance rides. Additionally, the recent studies on Arabian and thoroughbred horses have shown a very high degree of variation in the *ACTN3* gene in response to various conditions like breeding strategies and selection pressure [19].

It is worth mentioning that the molecular network of the *ACTN3* gene showed direct association with the *SH3RF2* gene, which is described as a cell anti-apoptotic factor. The previous studies have shown a significant downregulation of *SH3RF2* during intensive physical effort, concluding that apoptosis leads to damaged muscle fibers replacement by new and better suited-muscle fibers to exercise [15]. As both *SH3RF2* and *ACTN3* genes could be potentially used as genetic markers of endurance predispositions in horses, their influence at the molecular level can be assumed and may constitute the basis for further research. Moreover, the obtained association network has presented the *MIOX* gene as a molecular link between other analyzed genes. The *MIOX* gene encodes the myo-inositol oxygenase; To date, first and rate-limiting enzymes in the myo-inositol (MI) metabolism pathway have not been investigated in horses. The increase in MIOX enzyme activity is in proportion to serum glucose concentrations and plays an important role in carbohydrate metabolism [20]. This finding provides a new look at the *MIOX* gene and creates opportunities for further research in the context of understanding the endurance predispositions of horses.

The *KL* gene encodes the Klotho protein, a transmembrane β-glucuronidase (EC number 3.2.1.31) capable of hydrolysing steroid β-glucuronides, described for the first time in 1997 by Makoto Kuro-o et al. [21] The primary function of Klotho is to activate the ion channel TRPV5, providing control over the sensitivity of the organism to insulin. Moreover, it influences intracellular signaling pathways including p53/p21, cAMP, protein kinase C (PKC) and WNT signaling pathways, modulating Ca^2+^ and phosphate homeostasis [4,22]. These features have encouraged further studies and analyses about the role of *KL* in sports. In the study by Kim et al. (2015) [23] the Klotho protein was described as an aging suppressor, being a crucial molecule in aging processes and its overexpression resulted in longevity. In turn, the research performed on *KL*-deficient mouse model in endurance running strongly indicated that this factor can be a key modulator of muscle performance [5].

In horses, the *KL* gene was described by [7] where its significant downregulation in response to intense physical effort was depicted using the high-throughput RNA-seq screening method. The results indicated that the expression level of *KL* gene can be considered as an exhaustion biomarker, and a lower expression would be indicative of better endurance ability in equines.

The results obtained within the present study have confirmed the downregulation of the *KL* gene as well as its two isoforms—v202 and v203. The significant differences in the expression level before and after the 120 km ride were obtained for the total *KL* expression and v202 variant. Interestingly, the transcript abundance of the v203 isoform was the highest in every conditions in comparison to the others—the v203 isoform compared to v202 in both measurement time—before and after the ride showed a 45.5-fold and 96-fold change, respectively. While the downregulation of *KL* in terms of endurance exercise was confirmed and considered as preferred and beneficial, the expression of the v203 variant was relatively high. This fact may suggest another role of the Klotho protein encoded by v203 variant in endurance effort that has not been described so far. While recent studies have investigated and described the *KL* expression and role in mice model [24] and in humans [25], the results obtained in the present study, on the basis of field observations, demonstrated the possibility of carrying out further research on equines.

The endurance exercise is a complex issue considering the complexity of biochemical changes occurring in the organism. In the present study, we have shown that both *ACTN3* and *KL* genes come in different variants and expression levels differs between each other. Our results also suggest a significant interaction between particular groups of genes in terms of adaptation to physical exertion, where the *SH3RF2* and *MIOX* genes play a crucial role. Further research in this matter may result in a wider knowledge of that topic and in describing genetic markers predisposed to long-term exercise.

The main limitation of this study is the small research group and the homogeneity of the group (different age and differences in the conditions of transport to the competition venue). These limitations cannot be avoided in field studies, which on the other hand pose the unique possibility to examine sport horses in the reality of competitions.

## 5. Conclusions

The results have shown surprisingly high differences between different isoforms expression level. Both *KL* and *ACTN3* genes represent features that allow them to be used as genetic markers of endurance performance in the future. The correlation network analyses have shown that the *MIOX*, *SH3RH2* and *TNNI2* genes are significantly involved in endurance effort metabolism which opens a way for further research in this matter.

## Figures and Tables

**Table 1:** The primer sequences for genes with its isoforms and endogenous control.

Gene Symbol	Gene Name	References (Ensembl Database)	Primers Sequence	Amplicon Length
*ACTN3*	Actinin alpha 3, total variant	ENSECAG00000018961	F: CAGGCCTTCATCGACTTCAT R: GGAGAAGGCCACATAATCCA	243
*ACTN3-201*	Actinin alpha 3 v 201	ENSECAT00000021149.1	F: TTTCAGAGATCGTCGACGGG R: AGATGTCCTGGATGGCAAAG	78
*ACTN3-202*	Actinin alpha 3 v 202	ENSECAT00000021249.1	F: TTGCTCCTGGAGGTCATTTC R: CCGATGGACACCAGCTTAAC	135
*KL*	Klotho total variant	ENSECAG00000008185	F: ACCAAGAGAGATGACGCCAA R: GGCTCAGGAAGTCGACATAGA	306
*KL-202*	Klotho v 202	ENSECAT00000008327.3	F: CTCACATGAAGTTCCGCCAA R: GGCTCAGGAAGTCGACATAGA	184
*KL-203*	Klotho v 203	ENSECAT00000108964.1	F: CCAAGACAGGCTGAGAGTGT R: TCATGGAAGGTTTGGGCTCA	179
*B2M*	Beta-2-Microglobulin	ENSECAG00000000685	F: TGTCTTTCAGCAAGGACTGG R: CAAGCCTTCATGATGCTGGT	159

## Data Availability

The original contributions presented in the study are included in the article, further inquiries can be directed to the corresponding author.
